# IGFBP-2 in Critical Illness: A Prognostic Marker in the Growth Hormone/Insulin-like Growth Factor Axis

**DOI:** 10.3390/pathophysiology31040045

**Published:** 2024-11-01

**Authors:** Christos Savvidis, Eleni Kouroglou, Efthymia Kallistrou, Dimitra Ragia, Sofia Dionysopoulou, Georgios Gavriiloglou, Vasiliki Tsiama, Stella Proikaki, Konstantinos Belis, Ioannis Ilias

**Affiliations:** Department of Endocrinology, Hippokration Hospital, 11527 Athens, Greece; csendo@yahoo.gr (C.S.); eleni.kouroglou@gmail.com (E.K.); ekallist@gmail.com (E.K.); dimitraragia@gmail.com (D.R.); sofiadion88@gmail.com (S.D.); gavrilg@otenet.gr (G.G.); tsiama.vicky@gmail.com (V.T.); stellapro21@gmail.com (S.P.); kbelis_socadm@yahoo.gr (K.B.)

**Keywords:** IGFBP-2, critical illness, COVID-19, growth hormone/IGF axis, biomarker, sepsis, cytokines

## Abstract

Critical illness (CI) triggers complex disruptions in the growth hormone (GH)/insulin-like growth factor (IGF) axis, significantly affecting the dynamics of insulin-like growth-factor-binding proteins (IGFBPs). Among these, IGFBP-2 shows a sustained elevation during CI, which inversely correlates with serum levels of IGF-1, IGFBP-3, and the acid-labile subunit (ALS). Although IGFBP-2 does not directly interact with ALS, it may influence the availability of IGFs by competing with other IGFBPs for binding to IGF-1 and IGF-2. Research suggests that this persistent elevation of IGFBP-2 is largely driven by cytokine activity during CI, reflecting an adaptive response rather than a direct result of GH/IGF axis dysregulation. The clinical importance of IGFBP-2 is emphasized by its correlation with disease severity in conditions like sepsis and coronavirus disease 2019 (COVID-19), where its levels are markedly elevated compared to healthy controls and are similar to those observed in sepsis from various causes. Beyond its role in endocrine regulation, IGFBP-2 appears to play a part in metabolic and inflammatory pathways. Elevated IGFBP-2 levels have been linked to increased mortality and longer hospital stays, indicating its potential utility as a prognostic marker. Furthermore, measuring plasma IGFBP-2 may have other diagnostic applications, aiding in the assessment of CI when traditional biomarkers are inconclusive.

## 1. Introduction: Growth Hormone/Insulin-like Growth-Factor-Binding Protein Dynamics in Critical Illness

**Figure 1:**
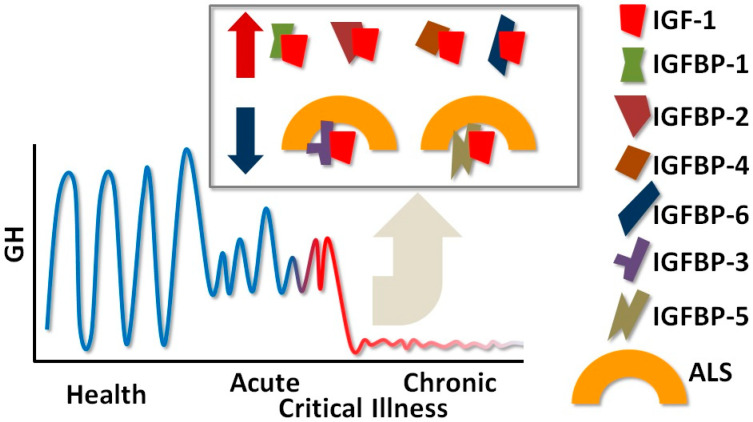
Growth hormone (GH), insulin-like growth factor (IGF-1), and IGF-binding proteins (IGFBP)-1 to -6 in critical illness (CI). In CI, the increased IGFBPs form binary complexes with IGF-1, prolonging the latter’s action, whereas the decreased IGFBPs form ternary complexes with IGF-1 and the acid-labile subunit (ALS); upwards facing arrow: increase, downwards facing arrow: decrease.

Critical illness (CI) induces multifaceted alterations in the GH/IGFBP (growth hormone/insulin-like growth-factor-binding protein) axis, characterized by a marked reduction in pulsatile GH secretion and increased GH resistance. This is typically accompanied by a variable response in the levels of insulin-like growth factor-1 (IGF-1), which further contributes to the disruption of the metabolic balance during CI. These shifts in the GH/IGFBP axis are not uniform, with differential expressions of various IGFBPs (insulin-like growth-factor-binding proteins) influencing the overall dysregulation of this axis during CI [1]. CI tends to follow a biphasic pattern. In the initial acute phase, there is a marked elevation in basal GH levels, while IGF-1 and IGFBP-3, along with its critical cofactor, the acid-labile subunit (ALS), show a marked decrease due to downregulation of GH receptors in peripheral tissues [2]. Conversely, levels of IGFBP-1, IGFBP-2, and IGFBP-6 increase during this phase, further inhibiting IGF-1’s biological activity and its role in cellular growth and metabolism [3] (Figure 1).

The acute phase of CI is characterized by the heightened release of cytokines such as Tumor Necrosis Factor-α (TNF-α), Interleukin-1 (IL-1), and IL-6. These cytokines, along with diminished IGF-1 levels, significantly elevate GH concentrations, promoting catabolic processes like increased lipolysis, which shifts energy metabolism away from anabolic functions [4,5]. In the chronic phase of CI, typically occurring after prolonged hospitalization of more than 7 to 10 days in the intensive care unit (ICU), GH secretion patterns become irregular and significantly reduced. This phase is also marked by further decreases in IGF-1, IGFBP-3, and ALS levels, reflecting the systemic adaptation to prolonged metabolic stress [2,6,7]. The decline in osteocalcin and leptin levels, which are markers of bone metabolism and energy balance, respectively, are associated with the reduction in IGF-1 and the IGFBP-3/IGFBP-5 complex during this phase [7] (Figure 1).

The experimental administration of GH-secretagogues, such as growth-hormone-releasing hormone (GHRH) and growth-hormone-releasing peptide (GHRP), during the chronic phase of CI has shown interesting results. More in detail: continuous infusion of these agents for up to two days can significantly increase GH pulsatile secretion by six to tenfold, along with increases in IGF-1, IGFBP-3, and ALS levels. This response suggests a potential restoration of peripheral GH responsiveness, which contrasts with the reduced IGFBP-3 levels observed in the acute phase [2,6,7]. These findings highlight the complex interplay between GH secretion and IGFBP dynamics, particularly in response to prolonged critical illness.

## 2. The Role of IGFBPs in Critical Illness

The somatotropic axis, which encompasses the IGF-binding proteins, has become an area of significant interest in the study of CI due to its profound effects on metabolism, catabolism, and nutritional status [8]. While the regulation of IGFBP-2 in CI is relatively well understood, substantial knowledge gaps remain concerning the regulation and potential clinical utility of other IGFBPs in this context. IGFBP-3, the most abundant IGFBP in circulation, binds a majority of the circulating IGF-1 in complex with ALS, thereby prolonging IGF-1’s half-life and enhancing its biological effects [9]. Besides its IGF-dependent actions, IGFBP-3 interacts with various components of the extracellular matrix and plasma membrane proteins, further modulating cell signaling pathways [10].

In the acute phase of CI, IGFBP-3 and ALS levels typically decline early in the course of illness and remain low due to peripheral GH resistance, reduced GH receptor expression, and impaired hepatic synthesis and release of IGF-1 and IGFBP-3 [8,11]. Several factors, including the severity of the disease, the duration of illness, and genetic variability, can influence IGFBP-3 levels. Additionally, the upregulation of serum protease activity, which increases during acute illness, is believed to contribute to the cleavage and degradation of IGFBP-3, further exacerbating its decline [12]. A reversal in these trends, including a reduction in protease activity and normalization of IGF-1 and IGFBP-3 levels, correlates with recovery from CI [12].

IGFBP-1 has emerged as an important regulator of IGF activity in acute CI. Its levels fluctuate in response to acute metabolic shifts and may predict impending metabolic changes. Elevated IGFBP-1 levels have been linked with a poorer catabolic status and increased mortality rates in critically ill patients [3]. Meanwhile, IGFBP-4 and IGFBP-6, whose levels increase during CI, may be upregulated in response to inflammatory cytokines. IGFBP-5, in contrast, declines during the chronic phase of CI, which, together with the fall in IGFBP-3, alters the distribution of IGF-IGFBPs complexes over ternary complexes that include ALS [11,13]. This redistribution may have significant implications for disease severity and metabolic homeostasis, as binary complexes are more bioavailable and may contribute to the heightened catabolic state observed in CI.

## 3. Functions of IGFBP-2

The regulation of IGFBP-2 is influenced by a range of endogenous and exogenous factors across both physiological and pathological settings. Endogenously, IGFBP-2 levels increase under certain conditions, such as fasting, or in response to the presence of GH and glucocorticoids. Additionally, various diseases like cancer—particularly glioblastoma and prostate cancer—as well as metabolic disorders such as type 2 diabetes (T2DM) and insulin resistance are associated with elevated IGFBP-2 levels. Exogenous factors also contribute to the upregulation of IGFBP-2; for example, administration of GH, glucocorticoids, and exposure to hypoxia can elevate IGFBP-2. Moreover, certain chemotherapeutic agents and environmental stressors, including nutrient deprivation, have been shown to stimulate IGFBP-2 production, underscoring its role in adaptive responses to cellular stress [14,15].

Post-translational modifications (PTMs) play a significant role in regulating IGFBP-2’s interaction with the IGF system. Phosphorylation, glycosylation, and proteolysis of IGFBP-2 and other IGF-binding proteins modulate their affinity for IGFs and their stability. Proteolytic cleavage of IGFBPs, for example, reduces their IGF-binding capacity, increasing the availability of free IGFs to interact with receptors and initiate signaling [16,17]. Furthermore, phosphorylation of IGFBP-2 can alter its affinity for IGF-1, affecting cellular proliferation and metabolic processes. Proteases like pregnancy-associated plasma protein-A (PAPP-A) can cleave IGFBPs, including IGFBP-4, which in turn diminishes their inhibitory effect on IGF signaling [16,17].

IGFBP-2’s functional complexity is further underscored by its structural features. Its nuclear localization signal (NLS) enables IGFBP-2 to enter the nucleus, where it can influence gene transcription and signaling pathways independent of IGF binding. Simultaneously, the arginine-glycine-aspartic acid (RGD) sequence allows IGFBP-2 to interact with integrins, affecting cellular processes like migration, survival, and tumor progression [14,15]. Proteolytic cleavage, primarily mediated by matrix metalloproteinases (MMPs) and other proteases, diminishes IGFBP-2’s affinity for IGFs in the tumor microenvironment, thereby increasing free IGFs and facilitating tumor cell proliferation and survival. Moreover, IGFBP-2 fragments generated through proteolysis possess unique biological properties that can influence cell adhesion, migration, and invasion [14,15]. These effects highlight the critical role of IGFBP-2 in modulating the tumor microenvironment and advancing cancer progression.

The impact of IGFBP-2 on IGF signaling remains a subject of debate. Conflicting evidence exists regarding its effect on IGF-1 binding to the IGF-1 receptor (IGF1R), with some studies suggesting it enhances IGF1R activation, while others indicate an inhibitory effect [17,18]. This dynamic regulation may also be influenced by cytokines such as TNF-α, IFN-γ, and interleukins, known to stimulate IGFBP-2 secretion, particularly in inflammatory conditions [19]. Hypoxia-inducible factor 1 (HIF-1), activated in low-oxygen environments, further upregulates IGFBP-2, promoting vascular endothelial growth factor (VEGF) production and angiogenesis [20,21].

IGFBP-2 plays a critical role in metabolism and growth regulation, often acting independently of IGF-1. It enhances insulin sensitivity and promotes glucose uptake in skeletal muscle cells while also improving hepatic insulin sensitivity [22,23]. Higher IGFBP-2 levels are linked to a favorable metabolic profile, characterized by reduced visceral fat accumulation and a decreased risk of obesity and insulin resistance [14,24,25]. Conversely, individuals with T2DM, obesity, or increased visceral fat tend to have lower IGFBP-2 levels compared to healthy lean subjects [26,27,28,29]. In clinical contexts marked by significant metabolic disruption, such as CI—where hepatic glucose production, insulin resistance, lipolysis, and protein catabolism are increased [30]—elevated IGFBP-2 levels may reflect compensatory mechanisms aimed at modulating these metabolic alterations.

In oncology, IGFBP-2 may assume a dual role, acting as an oncogene in a certain context and functioning as a tumor suppressor, depending on the cellular environment. For instance, IGFBP-2 can inhibit tumor growth by modulating IGF signaling or interacting with tumor suppressor proteins like phosphatase and tensin homolog (PTEN). However, in certain cancers, particularly when overexpressed, IGFBP-2 promotes tumor growth, invasion, and metastasis via IGF-independent mechanisms. These mechanisms often involve interactions with integrins such as αvβ3, which enhance cell migration and angiogenesis [14,15]. This context-dependent behavior of IGFBP-2 is a key factor in its varied effects across different tumor microenvironments. When dysregulated, IGFBP-2 can contribute to malignancy by promoting pro-tumorigenic pathways [14,15]. IGFBP-3, another member of the IGF-binding protein family, contrasts IGFBP-2 in its functions. While both regulate IGF activity, IGFBP-3 predominantly acts as a tumor suppressor by inhibiting IGF-1 and IGF-2 from activating IGF-1R, thus reducing cell proliferation and promoting apoptosis. In addition to its IGF-related actions, IGFBP-3 exerts IGF-independent effects, influencing processes like apoptosis, cell cycle regulation, and stress responses through interactions with nuclear receptors [14,15]. Conversely, IGFBP-2, while capable of binding IGFs, is frequently associated with tumorigenic behaviors, including increased migration, invasion, and survival of cancer cells, particularly via IGF-independent mechanisms. Elevated IGFBP-2 levels are often seen in cancers such as glioblastoma, prostate cancer, and ovarian cancer, where they contribute to increased metastasis and aggressiveness. In contrast, IGFBP-3 is often downregulated in cancer, leading to reduced tumor-suppressive effects [14,15]. Together, IGFBP-2 and IGFBP-3 exemplify the complexity of IGF regulation in cancer biology, with their distinct roles contributing to the multifaceted nature of tumor development.

Beyond oncology, elevated IGFBP-2 levels have been correlated with disease severity and mortality in conditions like heart failure and acute coronary syndrome [31,32]. Additionally, increased IGFBP-2 is noted in severe diseases such as diabetic kidney disease and lupus nephritis, reinforcing its potential as both a marker of disease severity and in disorders characterized by metabolic and inflammatory dysregulation [33,34].

## 4. IGFBP-2 in the Response to CI

CI significantly disrupts various endocrine pathways, including the GH/IGF axis, where IGFBP-2 plays a notable role in the body’s adaptive response [11]. During CI, most circulating IGFs are bound in ternary complexes with IGFBP-3, IGFBP-5, and the ALS. Initially, GH secretion increases with concurrent tissue resistance, but this shifts in prolonged illness, leading to decreased GH secretion and enhanced tissue sensitivity. This alteration contributes to a decline in IGFBP-3 and ALS levels [3]. Although IGFBP-2 does not directly interact with ALS, it may indirectly regulate IGF bioavailability by competing with other IGFBPs for IGF binding [11,35,36]. When total IGF levels exceed the binding capacity of IGFBP-3, IGFBP-2 takes on a more prominent role by binding excess free IGFs. In this way, IGFBP-2 helps control the availability of IGFs for signaling pathways that are vital for cellular growth and metabolism, making its role crucial during the metabolic and endocrine disruptions seen in CI [3,11].

IGFBP-2’s increased relevance during CI is closely tied to its interaction with IGFBP-3, the most abundant IGFBP in circulation. IGFBP-3 modulates IGF bioavailability by sequestering IGFs, preventing their interaction with IGF receptors, which are critical for cellular growth and differentiation. In the context of elevated IGFBP-2 levels, there is evidence that IGFBP-3 undergoes limited proteolysis, reducing its ability to sequester IGFs. This proteolysis, which may be exacerbated by higher levels of IGFBP-2, decreases the release of IGFs, thereby modulating their bioactivity [17,37]. This dynamic reciprocation between IGFBP-2 and IGFBP-3 highlights the complexity of IGF regulation in CI. The proteolysis of IGFBP-3 is a crucial process, particularly since IGFs, if released in excess, play a role in pathologies such as cancer, where they promote cellular proliferation [17,37]. IGFBP-2 may also influence protease activity, especially matrix metalloproteinases (MMPs) and pregnancy-associated plasma protein-A (PAPP-A), both of which mediate IGFBP-3 degradation. Elevated levels of IGFBP-2 can potentially alter these proteases’ activities, further influencing IGF [17,37].

The regulation of IGF signaling by IGFBP-2 and IGFBP-3 is largely determined by their relative affinities for IGF-1 and IGF-2. Under normal conditions, IGFBP-3, with its higher binding affinity, primarily sequesters both IGF-1 and IGF-2. However, during critical illness, when total circulating IGFs surpass IGFBP-3’s binding capacity, IGFBP-2 assumes a compensatory role by binding excess IGFs [11,35,36]. This shift becomes particularly important for IGF-2, for which IGFBP-2 has a higher affinity than IGF-1 [17,37]. This alteration in IGF-binding dynamics has significant downstream consequences. IGF-1 primarily signals through the IGF-1 receptor to stimulate growth, while IGF-2 can activate both IGF-1 and IGF-2 receptors. Therefore, increased IGFBP-2 binding to IGF-2 could lead to altered receptor activation and signaling pathways, which are especially important in CI and pathological states such as cancer, where IGFs play a role in promoting cell proliferation [17,37].

The availability of free IGFs—those not bound to IGFBPs—is crucial for their mitogenic and anti-apoptotic effects. In CI, as IGFBP-3’s capacity to bind IGFs becomes overwhelmed, IGFBP-2 helps regulate the remaining free IGFs, particularly IGF-2. This process alters the balance of free IGFs, subsequently influencing IGF signaling pathways that control vital physiological responses, including cell survival, metabolism, and repair [17,37]. Thus, the interplay between IGFBP-2 and IGFBP-3, alongside their regulation by proteases such as MMPs and PAPP-A, underpins a complex regulatory network that controls IGF signaling during critical illness. The increased importance of IGFBP-2 in this context emphasizes its role in modulating IGF bioavailability and signaling pathways, with broad implications for cellular growth, metabolism, and recovery from severe stress.

Elevated plasma IGFBP-2 levels have been consistently associated with CI, particularly in conditions such as sepsis and acute respiratory distress syndrome (ARDS) [38]. In these diseases, increased IGFBP-2 levels have been linked to poorer clinical outcomes, including higher mortality rates and longer ICU stays [1,3,39]. It is speculated that the rise in IGFBP-2 during CI reflects a compensatory response to the catabolic state induced by the illness, which is characterized by muscle wasting, insulin resistance, and disrupted glucose metabolism. This elevation of IGFBP-2 may also serve as an adaptive mechanism aimed at mitigating some of the metabolic disturbances inherent to CI.

## 5. Tentative Diagnostic Utility of IGFBP-2 in Infectious Diseases

The relationship between IGFBP-2 levels and infectious diseases has received increased attention in recent years, with research exploring its diagnostic and prognostic potential. In viral, bacterial, and parasitic infections, IGFBP-2 levels often fluctuate, potentially providing insights into the severity and progression of these diseases. For instance, in patients infected with Helicobacter pylori, IGFBP-2 levels are elevated, while both IGF-1 and IGF-2 levels are decreased [40]. Similarly, in individuals with HIV, plasma IGFBP-2 levels are positively correlated with IL-6, while cerebrospinal fluid (CSF) IGFBP-2 levels correlate with TNF-α levels [41], suggesting that IGFBP-2 could serve as a valuable biomarker for tracking disease progression in inflammatory and infectious diseases [41], particularly of the central nervous system [42].

IGFBP-2 also shows diagnostic potential in parasitic infections. In conditions such as echinococcosis and trichinellosis, circulating IGFBP-2 levels are significantly elevated; in toxoplasmosis, though, IGFBP-2 levels remain similar to those of healthy controls [43]. This suggests that specific parasitic infections may lead to distinct alterations in IGFBP-2 plasma levels, which could aid in diagnosing and differentiating between various parasitic diseases.

In bacterial infections, particularly in patients with E. coli-induced hemolytic uremic syndrome (HUS), IGFBP-2 levels are elevated and correlate with disease severity [44]. As HUS progresses, IGFBP-2 levels increase significantly, further highlighting its potential as a biomarker for infectious disease severity and progression. Additional research is needed to fully elucidate IGFBP-2’s diagnostic utility across a range of infectious diseases, but preliminary findings suggest that it holds promise as a tool for enhancing clinical decision-making, particularly in cases where traditional biomarkers are insufficient.

## 6. IGFBP-2 in Sepsis and COVID-19

Sepsis, a life-threatening condition characterized by dysregulated immune responses to infection, is associated with hypercatabolism and diminished IGF levels [12,45,46]. SARS-CoV-2 infection, responsible for COVID-19, has emerged as a major cause of sepsis in recent years. Survivors of severe COVID-19 often exhibit higher IGF-1 levels, while those with lower IGF-1 tend to have worse outcomes [47]. Interestingly, IGFBP-2 was shown to discriminate patients with COVID-19 from healthy subjects, serving as controls [48]. In critically ill patients, circulating IGFBP-2 levels can exceed the normal range, doubling or even tripling in severe cases [36,39,49,50,51]. Studies have demonstrated a threefold increase in plasma IGFBP-2 levels in septic patients compared to healthy controls, with the highest levels observed in those with septic shock, indicating a strong correlation between IGFBP-2 levels and disease severity [39].

In patients with COVID-19, elevated IGFBP-2 levels have been measured, particularly in non-survivors. However, the relationship between IGFBP-2 and COVID-19 is complex, as obese COVID-19 patients tend to exhibit lower IGFBP-2 levels compared to non-obese patients, necessitating further investigation into the interplay between metabolic factors and IGFBP-2 expression in different patient populations [49]. Overall, elevated IGFBP-2 levels in sepsis and COVID-19 point to its potential as a marker for disease severity and mortality risk.

## 7. Association of IGFBP-2 Levels with Mortality

IGFBP-2 plasma levels have been correlated with disease severity and outcomes. Research indicates that patients with systemic inflammatory response syndrome (SIRS) or sepsis exhibit markedly elevated IGFBP-2 levels compared to healthy controls, with these elevations correlating with the need for dialysis, disease severity, and ultimately mortality [39].

High IGFBP-2 concentrations have also been documented in ICU patients, indicating a potential link to increased mortality risk [3]. Research on cardiovascular diseases has highlighted the prognostic value of IGFBP-2 as well; for example, Barutaut et al. identified plasma IGFBP-2 levels as predictors of mortality in heart failure patients [32]. Additionally, elevated IGFBP-2 levels in acute coronary syndrome patients correlate with low ejection fraction and a heightened risk of major cardiovascular complications [31]. Moreover, large-scale studies have established a connection between high IGFBP-2 levels and metabolic dysfunction within the general population. Interestingly, these studies suggest an age-related increase in plasma IGFBP-2 levels, particularly in individuals over 50, which parallels the development of insulin resistance and may serve as a mortality predictor after adjusting for insulin sensitivity [52]. In the context of COVID-19, studies have found a clear association between elevated serum IGFBP-2 levels and fatal outcomes in hospitalized patients [49]. Overall, the association between IGFBP-2 levels and mortality across multiple diseases emphasizes its potential role as a prognostic biomarker.

## 8. Discussion

**Figure 2:**
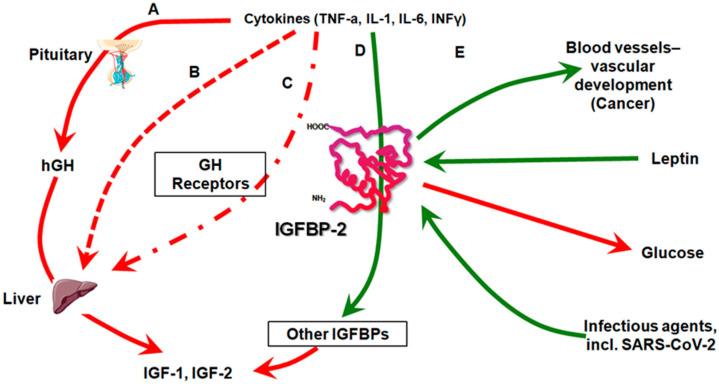
The role of IGFBP-2 in cytokine regulation of the GH/IGF axis: central influences on cancer, metabolism, and infectious diseases. A. Cytokines and GH Secretion: Cytokines modulate the secretion of growth hormone (GH) from the pituitary gland. During inflammatory responses, there is typically a reduction in the pulsatility and overall secretion of GH (indicated by the red solid line), signifying a suppressive effect. B. Impact on Liver Function: While GH normally stimulates IGF-1 production in the liver, cytokines can impair this process, leading to decreased levels of circulating IGF-1 (red square dotted line), representing a negative regulatory effect. C. GH Receptor and Signaling: Cytokines may decrease the expression and signaling efficiency of GH receptors, reducing tissue sensitivity and efficacy, a phenomenon known as GH resistance (red dash dotted line). D. IGFBPs Regulation: Cytokines alter the levels of insulin-like growth-factor-binding proteins (IGFBPs), like IGFBP-1 and IGFBP-2. These proteins regulate the availability and activity of IGFs by binding and preventing their interaction with receptors (green solid line), showcasing a positive regulatory mechanism. E. IGFBP-2’s Diverse Roles: Cancer: IGFBP-2 promotes cancer progression by enhancing tumor growth and vascular development, partly through stimulating angiogenesis mediated by hypoxia-inducible factors and VEGF. Metabolism: Interacts with leptin to regulate insulin sensitivity and glucose metabolism. Higher IGFBP-2 levels are linked to reduced adipogenesis and better glucose control, potentially protecting against obesity and type 2 diabetes. Critical Illnesses: In conditions like COVID-19, elevated IGFBP-2 levels correlate with increased disease severity, highlighting its potential as a prognostic biomarker (Green lines signify positive regulatory effects that enhance or promote physiological functions. Red lines indicate negative regulatory effects that suppress or diminish physiological functions).

The significance of IGFBP-2 in the pathophysiology of CI, particularly in sepsis and COVID-19, is increasingly recognized. Elevated levels of IGFBP-2 correlate inversely with serum IGF-1, suggesting a compensatory mechanism in response to the systemic inflammatory state typical of CI [39]. Its consistent association with disease severity and increased mortality highlights its potential as a prognostic biomarker [32]. Furthermore, the rise in IGFBP-2 during CI may reflect an adaptive response to the cytokine storm, common in severe infections and septic conditions, including those seen in COVID-19 (Figure 2). Thus, IGFBP-2 serves not only as a marker of disease severity but also as an indicator of inflammatory status and immune response [38].

The differing trajectories of IGFBP-2 compared to other IGFBPs during CI suggest a role that extends beyond its classical function within the GH/IGF axis [50]. Emerging evidence also supports its diagnostic potential in complex clinical scenarios where traditional biomarkers may fall short. Elevated IGFBP-2 levels in sepsis and COVID-19 patients compared to other CI etiologies could aid in differential diagnosis [50].

## 9. Conclusions

The course of IGFBP-2 in CI, particularly in the contexts of sepsis andCOVID-19, points to its significant role as a biomarker of disease severity. The strong correlation between elevated IGFBP-2 levels and increased mortality, coupled with its involvement in modulating inflammatory and immune responses, highlights the urgent need for continued research in this area. Further investigations into the mechanisms underlying IGFBP-2 dynamics in critical illness may yield valuable insights that may enhance diagnostic approaches for affected patients.

## Data Availability

Not applicable.
